# Chlorogenic acid improves glucose tolerance, lipid metabolism, inflammation and microbiota composition in diabetic db/db mice

**DOI:** 10.3389/fendo.2022.1042044

**Published:** 2022-11-17

**Authors:** Yongwang Yan, Qing Li, Ling Shen, Kangxiao Guo, Xu Zhou

**Affiliations:** ^1^ Pharmaceutical College, Changsha Health Vocational College, Changsha, China; ^2^ Department of Pathology, Changsha Health Vocational College, Changsha, China; ^3^ National Engineering Laboratory for Rice and By-Product Deep Processing, College of Food Science and Engineering, Central South University of Forestry and Technology, Changsha, China; ^4^ Department of Spleen, Stomach and Liver Diseases, Affiliated Hospital of Hunan Academy of Traditional Chinese Medicine, Changsha, China

**Keywords:** chlorogenic acid, hyperglycemia, hyperlipidemia, inflammation, oxidative stress

## Abstract

**Introduction:**

Chronic and acute chlorogenic acid (CGA) can improve glucose tolerance (GT) and insulin sensitivity (IS). However, whether acute administration of CGA has beneficial effects on hepatic lipid metabolism and cecal microbiota composition remains unclear.

**Methods:**

In the current study, diabetic db/db mice were administered CGA or metformin, and db/m mice were used as controls to explore the effects of CGA on hepatic lipid metabolism, including fatty acid oxidation and transportation and triglyceride (TG) lipolysis and synthesis. Moreover, alterations in the inflammatory response and oxidative stress in the liver and gut microbe composition were evaluated.

**Results:**

The results showed that CGA decreased body weight and improved glucose tolerance and insulin resistance, and these effects were similar to those of metformin. CGA decreased hepatic lipid content by increasing the expression of *CPT1a* (carnitine palmitoyltransferase 1a), *ACOX1* (Acyl-CoA oxidase 1), *ATGL* (adipose triglyceride lipase), and *HSL* (hormone-sensitive lipase) and decreasing that of *MGAT1* (monoacylglycerol O-acyltransferase 1), *DGAT1* (diacylglycerol O-acyltransferase), *DGAT2, CD36*, and *FATP4* (fatty acid transport protein 4). Additionally, CGA restored the expression of inflammatory genes, including *TNF-α* (tumor necrosis factor-alpha), *IL-1β (interleukin-1beta), IL-6*, and *IL-10*, and genes encoding antioxidant enzymes, including *SOD1* (superoxide dismutases 1), *SOD2* (superoxide dismutases 2), and *GPX1* (glutathione peroxidase 1). Furthermore, CGA improved the bacterial alpha and beta diversity in the cecum. Moreover, CGA recovered the abundance of the phylum Bacteroidetes and the genera *Lactobacillus, Blautia*, and *Enterococcus*.

**Discussion:**

CGA can improve the antidiabetic effects, and microbes may critically mediate these beneficial effects.

## Introduction

Chlorogenic acid (CGA), a member of the hydroxycinnamic acid family, is abundant in many plants, including coffee beans, apples, tea, and tobacco leaves (1). CGA has been proven to be one of the most promising phenolic acids, serving as either a food additive or nutraceutical due to its efficacy in alleviating oxidative stress, the inflammatory response, and glucose and lipid metabolism disorders (1–3). Based on these effects, the use of CGA for treating metabolic syndromes, such as type 2 diabetes mellitus and cardiovascular diseases, has been widely studied (4). Human and animal studies have confirmed that CGA can dramatically lower total cholesterol and total triglyceride levels (5, 6). In overweight patients, taking CGA twice a day can improve blood glucose, insulin sensitivity and other metabolic parameters (7). In high-fat diet (HFD)-fed mice, CGA exerts its protective effect on obesity and related metabolic syndrome by regulating gut microbiota structure, diversity, and changes in relative abundance at the phylum to genus levels (8). AMPK (AMP-activated protein kinase) signaling and gut microbes are involved in the anti-diabetic and anti-lipidemic effects of CGA (9). However, the detailed mechanisms require further investigation.

Gut microbes are considered to be important health regulators, and dietary components are one of the major factors determining the composition of the gut microbiota (10, 11). Approximately 30% of dietary CGA is absorbed in the small intestine (12, 13), indicating that CGA can reach the large intestine and exert direct effects on microbes. Numerous studies have demonstrated the modulatory effects of CGA on the diversity and composition of the microbiota. CGA can decrease the abundance of *Lachnospiraceae*, *Ruminococcaceae*, *Desulfovibrionaceae*, *and Erysipelotrichaceae* and increase that of *Lactobacillaceae* and *Bacteroidaceae* (14). Significantly, CGA improved the abundance of beneficial bacteria, including *Ruminococcaceae UCG-005, Akkermansia*, *Christensenellaceae R-7_group*, and *Rikenellaceae RC9_gut_group* (15) and short-chain fatty acid producers such as *Faecalibaculum*, *Romboutsia*, *Mucispirillum*, and *Dubosiella* (16). Meanwhile, CGA reduced the abundance of bacteria involved in inflammation, such as *Sutterella* species and those that cause intestinal dysfunction, such as *Escherichia coli* and other LPS-producers (17, 18). These changes in microbiota composition, induced by CGA, mediate the positive effects of CGA on metabolic disorders. However, whether CGA administration can recover the microbiota composition of diabetic mice has not been fully determined.

A previous study reported the acute effects of CGA on glucose tolerance (GT), insulin sensitivity (IS), gluconeogenesis, and fatty acid synthesis in Lepr^db^ diabetic (db/db) mice (11). However, whether acute treatment with CGA affects fatty acid oxidation, inflammatory responses, and microbiota composition remains to be explored. Consequently, we investigated the acute effects of CGA on lipid metabolism and the inflammatory and antioxidative status in db/db diabetic mice. Furthermore, the involvement of intestinal microbes in the beneficial effects of CGA has been elucidated

## Materials and methods

### Animal experiments

Eighteen male diabetic db/db mice and six male db/m mice were provided by Gene&Peace Biotech Co., Ltd (Guangzhou, China). The db/db mice were assigned to the following treatment groups: (1) DB group, animals were orally gavaged with 0.1 mL PBS once a day; (2) CA group, animals were gavaged with 0.25 g CGA/kg body weight (BW) once a day; (3) MET, animals were gavaged with 0.25 g metformin/kg BW once a day. The concentration of CGA used was based on our preliminary experiment. The db/m mice were assigned as the control (CON). CGA and metformin were purchased from Sigma (Shanghai, China). The experiment lasted 18 days. All mice had free access to water and feed. Feed intake and BW were recorded during the experiment. Animals were treated humanely, using approved procedures in accordance with the guidelines of Changsha Health Vocational College. The study was approved by the Institutional Animal Care and Use Committee of Changsha Health Vocational College (2021053).

### Oral GT and IS tests

Fasting blood glucose was tested every 7 days, and it was significantly decreased on Day 14 after treatment with CGA or metformin. Then, oral GT and IS tests were performed. After fasting for 6 h, mice were either orally gavaged with a dose of 2.0 g glucose or intraperitoneally injected with a dose of 0.65 U insulin/kg BW. Blood was obtained from the tail vein, and the glucose concentration was analyzed at 0, 30, 60 and 120 min.

### Sample collection

First, blood was obtained from the retro-orbital sinus and serum samples were separated. Then, after cervical dislocation, liver, inguinal and epididymal adipose tissue were separated and weighed. Furthermore, liver samples were either fixed in formaldehyde solution or stored at −80°C for analysis of gene expression. Additionally, cecum content was obtained for the determination of gut microbiota.

### Serum biochemistry assay

The contents of glucose, high-density lipoprotein cholesterol (HDL-C), low-density lipoprotein cholesterol (LDL-C), total cholesterol (TC) and triglycerides (TGs), and the activities of alanine aminotransferase (ALT) and aspartate aminotransferase (AST) were determined by using commercial kits (Meimian, Nantong, China) as previously described (19).

### Hepatic histological and lipid content analyses

Fixed liver samples were paraffin embedded and sectioned into 8-μm thick sections; then, the samples were either subjected to H&E (hematoxylin–eosin) staining for histological observation or Oil Red O staining for the determination of lipid content as previously described (20).

### RT‐qPCR

Total RNA was extracted using Trizol reagent (Invitrogen, Shanghai, China) and DNA was removed after treatment with DNase I (Takara, Beijing, China). Then, cDNA was synthesized using a RevertAid Reverse Transcription Kit (Thermo Scientific) as previously described (21). RT‐qPCR was carried out by using SYBR Green mix (Applied Biosystems). The primers used for qPCR are shown in **Supplementary Table 1**.

### Gut microbiota profiling

DNA was extracted with cecum contents, and bacterial 16S rRNA gene sequences (V3–V4 regions) were amplified. PCRs were carried out with Phusion High-Fidelity PCR Master Mix (Thermo Fisher). Then, MiSeq Illumina sequencing was performed with products with a length of 400-450 bp. The Greengenes database with the RDP algorithm was used to analyze representative OTUs.

### Statistical analysis

Statistical analysis was performed using one-way ANOVA with Tukey’s *post hoc* test using SPSS 18.0. All data were expressed as the means ± SEM, and significant differences were confirmed when *P* < 0.05.

## Results

### Effects of chlorogenic acid on body weight, feed intake and adipose tissue and liver weight in db/db mice

As shown in **Table 1**, the BW of db/db diabetic mice was significantly higher than that of db/m mice at the beginning of the experiments. The BW of mice in the CA and MET groups was significantly lower than that in the DB group, whereas it remained significantly higher than that in the CON group on day 7, 14 and 18. Mice in the CA and MET group had lower daily feed intake than those in the DB group, whereas they had higher daily feed intake than those in the CON group. As shown in **Table 2**, the weights of inguinal fat and liver in mice in the CA and MET groups were significantly lower than those in the DB group, whereas they were significantly higher than those in the CON group. Epididymal fat weight was significantly lower in mice in the CON group than in the other three groups.

**Table 1:** Effects of chlorogenic acid on body weight and feed intake in db/db mice.

	CON	DB	CA	MET
Body weight (Day 0), g	23.50 ± 2.15^b^	38.20 ± 0.98^a^	39.00 ± 0.80^a^	39.00 ± 1.00^a^
Body weight (Day 7), g	24.00 ± 2.17^b^	41.78 ± 0.89^a^	40.55 ± 0.82^a^	40.48 ± 1.88^a^
Body weight (Day 14), g	22.55 ± 1.97^c^	41.27 ± 0.97^a^	38.98 ± 0.70^b^	38.63 ± 1.08^b^
Body weight (Day 18), g	23.33 ± 1.68^c^	41.30 ± 1.11^a^	37.93 ± 0.65^b^	37.90 ± 0.95^b^
Daily feed intake, g	3.28 ± 0.17^c^	5.62 ± 0.31^a^	4.69 ± 0.36^b^	4.41 ± 0.19^b^

^a,b,c^ Groups that share the same superscript letters are not significantly different from each other (P < 0.05). Data are expressed as the means ± SEMs, n=6.

**Table 2:** Effects of chlorogenic acid on adipose tissue and liver weight in db/db mice.

	CON	DB	CA	MET
Inguinal fat weight, g	0.146 ± 0.014^c^	1.617 ± 0.062^a^	1.239 ± 0.094^b^	1.127 ± 0.068^b^
Epididymal fat weight, g	0.118 ± 0.010^b^	1.541 ± 0.053^a^	1.43 ± 0.062^a^	1.504 ± 0.042^a^
Liver weight, g	1.087 ± 0.072^c^	2.595 ± 0.074^a^	2.450 ± 0.072^b^	2.407 ± 0.089^b^

^a,b,c^ Groups that share the same superscript letters are not significantly different from each other (P < 0.05). Data are expressed as the means ± SEMs, n=6.

### Effects of chlorogenic acid on GT and IS in db/db mice

**Figure 1:**
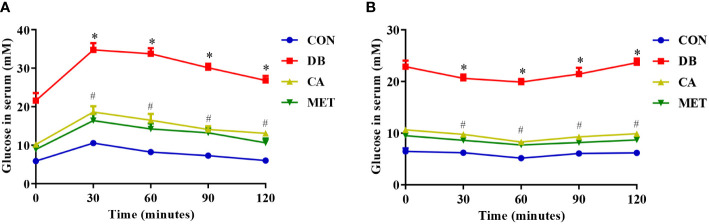
Effects of chlorogenic acid on glucose tolerance and insulin resistance in diabetic db/db mice. **(A)** Glucose tolerance; **(B)** Insulin resistance. Data are expressed as the means ± SEMs, n=6. *P < 0.05 (significant difference between mice in the DB group and mice in the other groups); ^#^ *P* < 0.05 (significant difference between mice in the CON group and mice in the CA and MET groups).

According to **Figure 1**, either chlorogenic acid or metformin improved GT and IS in db/db mice, whereas all the db/db mice still showed lower GT and IS than the db/m mice.

### Effects of chlorogenic acid on serum biochemical indicators in db/db mice

As shown in **Table 3**, glucose and TG contents were significantly lower in mice in the CA and MET groups than in the DB group, whereas they were significantly higher than those in the CON group. Compared with those in the DB group, mice in the other three groups had significantly lower TC and LDL-C contents, and higher HDL-C contents. The activities of AST and ALT were significantly lower in mice in the CA and MET groups than in the DB group, whereas they were significantly higher in mice in the CA group than in the CON group.

**Table 3:** Effects of chlorogenic acid on serum biochemical indicators in db/db mice.

Index	CON	DB	CA	MET
Glucose, mM	5.83 ± 0.57^c^	23.67 ± 1.09^a^	9.45 ± 0.64^b^	8.73 ± 0.77^b^
TG, mM	0.78 ± 0.22^c^	2.02 ± 0.34^a^	1.23 ± 0.22^b^	1.17 ± 0.18^b^
TC, mM	1.88 ± 0.32^b^	5.68 ± 0.35^a^	2.13 ± 0.45^b^	1.83 ± 0.33^b^
LDL-C, mM	0.36 ± 0.05^c^	1.62 ± 0.11^a^	0.56 ± 0.08^b^	0.46 ± 0.12^bc^
HDL-C, mM	2.32 ± 0.14^b^	1.65 ± 0.16^a^	2.24 ± 0.20^b^	2.31 ± 0.15^b^
AST, U/L	22.6 ± 3.0^c^	54.2 ± 4.6^a^	28.3 ± 5.8^b^	27.5 ± 4.1^bc^
ALT, U/L	22.7 ± 2.9^d^	99.6 ± 8.7^a^	43.1 ± 6.3^b^	31.7 ± 5.6^c^

^a,b,c,d^ Groups that share the same superscript letters are not significantly different from each other (P < 0.05). Data are expressed as the means ± SEMs, n=6.

### Effects of chlorogenic acid on hepatic morphology and lipid content in db/db mice

**Figure 2:**
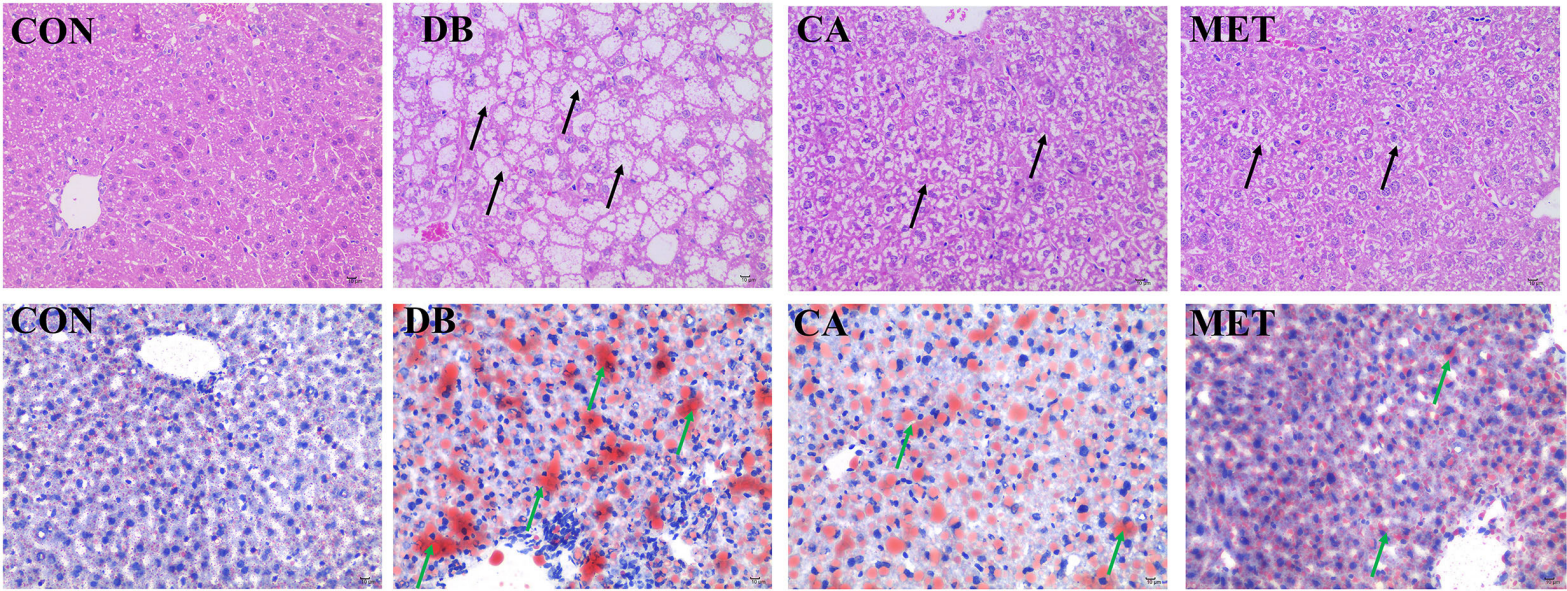
Effects of chlorogenic acid on liver morphology and lipid accumulation in diabetic db/db mice. Upper panel, representative results of H&E staining (400×); Lower panel, representative results of Oil red O staining (400×). Black arrows, damaged hepatocytes; green arrows, lipid droplets.

According to **Figure 2**, H&E staining showed that liver morphology was significantly damaged (microvesicular fatty change) in db/db diabetic mice, whereas the administration of chlorogenic acid and metformin both alleviated the impairment, although the morphology was still not returned to normal. Oil red O staining showed that db/db diabetic mice had higher lipid accumulation in the liver, whereas the administration of chlorogenic acid and metformin both decreased hepatic lipid accumulation.

### Effects of chlorogenic acid on expression of lipid metabolism-related genes in live db/db mice

**Figure 3:**
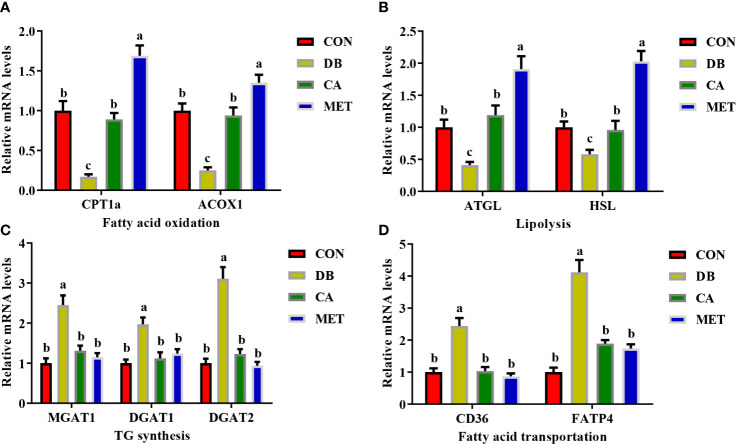
Effects of chlorogenic acid on the expression of genes involved in lipid metabolism in the livers of diabetic db/db mice. **(A)** Expression of genes involved in fatty acid oxidation; **(B)** Expression of genes involved in lipolysis; **(C)** Expression of genes involved in TG synthesis; **(D)** Expression of genes involved in fatty acid transportation. Data are expressed as the means ± SEMs, n=6. ^a,b,c^ Groups that share the same superscript letters are not significantly different from each other (*P* < 0.05).

The expression of genes including *CPT1a*, *ACOX1* (**Figure 3A**), *ATGL* and *HSL* (**Figure 3B**) was significantly lower in mice in the CON and CA groups than in the MET group, whereas they were significantly higher than those in the DB group. Compared with mice in the DB group, mice in the other three groups had significantly lower expression of genes involved in TG synthesis (*MGAT1*, *DGAT1* and *DGAT2*) (**Figure 3C**) and fatty acid transport (*CD36* and *FATP4*) (**Figure 3D**).

### Effects of chlorogenic acid on the expression of inflammation- and antioxidant ability-related genes in live db/db mice

**Figure 4:**
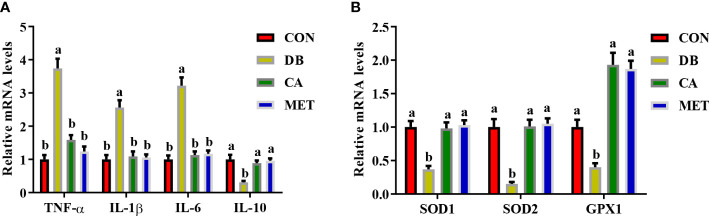
Effects of chlorogenic acid on the expression of genes encoding inflammatory cytokines and antioxidant enzymes in the livers of diabetic db/db mice. **(A)** Expression of genes encoding inflammatory cytokines; **(B)** Expression of genes encoding antioxidant enzymes. Data are expressed as the means ± SEMs, n=6. ^a,b^ Groups that share the same superscript letters are not significantly different from each other (*P* < 0.05).

Compared with mice in the DB group, mice in the other three groups had significantly lower expression of genes (*IL-1β*, *TNF-α* and *IL-6*) encoding proinflammatory cytokines and higher expression of the *IL10* gene encoding an anti-inflammatory cytokine (**Figure 4A**). Compared with mice in the DB group, mice in the other three groups had significantly higher expression of genes including *SOD1*, *SOD2* and *GPX1* (**Figure 4B**).

### Effects of chlorogenic acid on cecal microbiota composition in db/db mice

**Figure 5:**
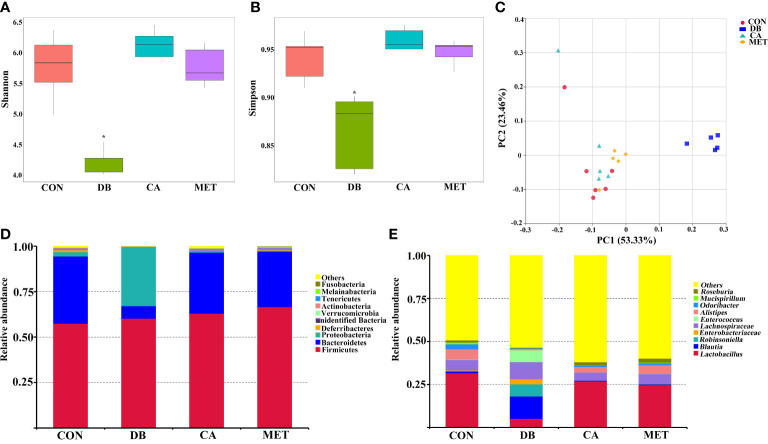
Effects of chlorogenic acid on cecal microbe composition in diabetic db/db mice. **(A)** Shannon index; **(B)** Simpson index; **(C)** PCoA plot of the microbiota based on an unweighted UniFrac metric; **(D)** Relative abundance of predominant bacteria at the phylum level; **(E)** Relative abundance of predominant bacteria at the genus level. *P < 0.05 (significant difference between mice in the DB group and mice in the other groups).

The alpha diversity of the microbial community, as indicated by the Shannon and Simpson index (**Figures 5A, B**), was significantly higher in mice in the CA and MET groups than in the DB group, whereas there was no significant difference among mice in the CA, MET and CON groups. Principal coordinates analysis (PCoA) showed that mice in the DB group were clearly separated from those in the other treatment groups (**Figure 5C**). Firmicutes, Proteobacteria and Bacteroidetes were the most abundant microbes at the phylum- level (**Figure 5D**). Compared with mice in the other groups, Bacteroidetes abundance was lower while Proteobacteria abundance was higher in mice of the DB group. Compared with mice in the other groups, *Lactobacillus* abundance at a genus-level taxonomy was lower while *Blautia*, *Robinsoniella* and *Enterococcus* abundance were higher in mice of the DB group (**Figure 5E**).

## Discussion

Db/db mice are commonly used models for studying diabetes. Chronic administration of a relatively low CGA concentration or acute administration of a high concentration in db/db mice improved GT and IS (22, 23). In the present study, CGA alleviated hyperglycemia, and hyperlipidemia and decreased hepatic lipid overaccumulation in db/db mice. Moreover, our results suggest that CGA alleviates the inflammatory response and enhances antioxidant activity. Furthermore, CGA improved microbiota diversity and recovered their composition in the cecum of db/db mice.

CGA can decrease lipid accumulation in obese individuals. In the present study, short-term administration of CGA reduced body weight, adipose tissue, and liver weight in db/db mice, although their weight was not comparable to that of control db/m mice. Previous studies have focused on the effects of CGA on gluconeogenesis and fatty acid synthesis (24–26), while our results suggest that CGA enhances hepatic lipolysis and reduces TG synthesis and fatty acid transportation in the liver of db/db mice. In addition, CGA improved the expression of genes involved in hepatic lipid metabolism in high-fat diet (HFD)-fed mice (27). These results suggest that CGA improves hepatic lipid metabolism in animal models of lipid metabolic disorders. Hepatic lipid overaccumulation is usually associated with activated inflammatory responses (28–30). Our results suggest that CGA alleviates inflammation, similar to a previous study in mice with HFD-induced obesity (31). HFD resulted in an imbalance in the oxidative status, which always accompanied by inflammation, and CGA administration recovered the antioxidant status in rats. The results confirmed the antioxidative ability of CGA, as indicated by the increased expression of genes encoding enzymes such as SOD and GPX (32, 33). Although our results showed the anti-inflammatory and anti-lipidemic effects of CGA in db/db mice, the specific targets of CGA and the possible signaling pathways that regulate the alteration in inflammation and lipid metabolism remain to be explored.

The gut microbiota can influence the differentiation and apoptosis of intestinal epithelial cells and positive regulation of the gut microbiota is conducive to alleviating metabolic syndromes (34–36). Some short-chain fatty acids among the metabolites of gut microbiota are capable of improving diet-induced obesity and insulin resistance (37). Several studies have demonstrated that the beneficial role of CGA is positively related to gut microbiota regulation (18, 38). For instance, CGA could increase the contents of *Bifidobacterium* and reduce *Escherichia coli* in NAFLD mice (17). CGA also exerts its antiobesity effects by ameliorating HFD-induced gut microbiota dysbiosis by inhibiting the growth of Desulfovibrionaceae, Ruminococcaceae, Lachnospiraceae, and Erysipelotrichaceae, and raising the growth of Bacteroidaceae, Lactobacill*aceae* (14, 39). However, studies have also demonstrated that CGA- treated mice showed microbial structures similar to those of the control group (40–43). According to these sudies and our results, we speculated that CGA could regulate gut microbiota structure under different pathological conditions in different ways. In our study, we found that CGA or metformin increased the α-diversity of the gut microbiota in db/db mice. Additionally, the β-diversity, as indicated by the PCoA, showed that mice administered CGA or metformin were similar to control mice but different from untreated db/db mice. These effects of CGA on the improvement of gut microbiota diversity were consistent with previous results using different models (44, 45). HFD-induced obesity and glucose and lipid metabolism disorders are often accompanied by increased abundance of Firmicutes and decreased abundance of Bacteroidetes and Proteobacteria (16). However, our results only showed similar results that Bacteroidetes abundance was decreased, whereas Firmicutes abundance was not changed and Proteobacteria abundance was increased in db/db mice. Another study showed that db/db mice did not show the alterations mentioned above when compared to wild-type m/m mice (46). These results suggest that different models of diabetic mice exhibit inconsistent changes in microbiota composition. Nevertheless, CGA restored the structure of the gut microbiota, and these effects were similar to those of metformin.

The most significant change at the genus level after CGA administration was the recovery of *Lactobacillus* abundance. This result suggested that CGA exerted beneficial effects, as most *Lactobacillus* species are considered to be beneficial microbes (47, 48). Surprisingly, *Blautia* abundance significantly increased in db/db mice, whereas CGA administration reduced this abundance. *Blautia* is a functional genus with probiotic properties, including improved metabolic function and host health (49). We speculated that the recovery of *Blautia* abundance by CGA could be an indirect effect and that *Blautia* may not be the critical genus mediating the beneficial effects of CGA. However, the underlying mechanisms need to be elucidated further. Additionally, the abundance of *Robinsoniella*, a spore-forming genus, was significantly increased in db/db mice, whereas CGA administration restored it. Alterations in the abundance of *Robinsoniella* are responsible for causing diseases such as nonalcoholic fatty liver and colitis (50, 51), indicating its role in lipid metabolism and inflammation. CGA administration notably decreased the abundance of *Enterococcus*, an important pathogen, when it reached a high density (24). These results indicate the modulatory effects of CGA on dysbiosis of the intestinal microbiota in diabetic db/db mice. However, the changes in microbes that contribute to the beneficial effects of CGA, especially the lipid-lowering effects, need to be further elucidated.

In conclusion, our results suggest that the acute administration of CGA improves GT and IS in diabetic db/db mice. CGA improved lipid metabolism by enhancing fatty acid oxidation and TG lipolysis and reducing TG synthesis and fatty acid transportation in the liver. Moreover, CGA alleviated the hepatic inflammatory response and oxidative stress. Significantly, CGA improved the diversity of the microbiota and promoted the recovery of microbiota composition. These data suggest that acute CGA treatment aids the anti-diabetic effect, and microbes may mediate these effects. Our results indicate that gut microbial alterations should be carefully monitored during the use of CGA in metabolic syndromes.

## Data availability statement

The data presented in the study are deposited in the NCBI repository, accession number PRJNA891362.

## Ethics statement

The animal study was reviewed and approved by Institutional Animal Care and Use Committee of Changsha Health Vocational College.

## Author contributions

YY and XZ designed the experiments. YY, QL, LS and KG performed the experiments, analyzed the data, and drafted the manuscript. XZ revised the manuscript. All authors contributed to the article and approved the submitted version.

## Funding

This work was supported by the Changsha Municipal Natural Science Foundation (grant number kq2014029) and Natural Science Foundation of Hunan Province (2022JJ60111).

## Conflict of interest

The authors declare that the research was conducted in the absence of any commercial or financial relationships that could be construed as a potential conflict of interest.

## Publisher’s note

All claims expressed in this article are solely those of the authors and do not necessarily represent those of their affiliated organizations, or those of the publisher, the editors and the reviewers. Any product that may be evaluated in this article, or claim that may be made by its manufacturer, is not guaranteed or endorsed by the publisher.

## Supplementary material

Supplementary Table 1 Primer sequences. The table provided the sequences of primers used for RT-qPCR analysis.
