# Supplementary material for: Chlorogenic acid improves glucose tolerance, lipid metabolism, inflammation and microbiota composition in diabetic db/db mice
Source: Front Endocrinol (Lausanne). 2022 Nov 17;13:1042044. doi: 10.3389/fendo.2022.1042044 (PMC9714618; doi:10.3389/fendo.2022.1042044)
Supplement: Supplementary Table 1 — Primer sequences. The table provided the sequences of primers used for RT-qPCR analysis. [file Table_1.docx]

Supplementary Table 1 Primer sequences

| Gene | 5’-3’ Primer sequence |
| --- | --- |
| *SOD1* | F: ATTGGCCGTACAATGGTGGT |
|  | R: ATCCCAATCACTCCACAGGC |
| *SOD2* | F: GTAGGGCCTGTCCGATGATG |
|  | R: CGCTACTGAGAAAGGTGCCA |
| *GPX1* | F: ACAGTCCACCGTGTATGCCT |
|  | R: ACCAGGTCGGACGTACTTGA |
| *IL-1β* | F: TGCCACCTTTTGACAGTGATG |
|  | R: AAGGTCCACGGGAAAGACAC |
| *IL-6* | F: CCTCTCTGCAAGAGACTTCCAT |
|  | R: AGTCTCCTCTCCGGACTTGT |
| *TNF-α* | F: ATGAGAAGTTCCCAAATGGC |
|  | R: CTCCACTTGGTGGTTTGCTA |
| *CPT1a* | F: CTCCGCCTGAGCCATGAAG |
|  | R: CACCAGTGATGATGCCATTCT |
| *ACOX1* | F: TTGTTGTCCCTATCCGTGAGA |
|  | R: GGCCGATATCCCCAACAGT |
| *ACADM* | F: TGCTCGCAGAAATGGCGATGA |
|  | R: CAATGTGCTCACGAGCTATGA |
| *ATGL* | F: ATATCCCACTTTAGCTCCAAGG |
|  | R: CAAGTTGTCTGAAATGCCGC |
| *HSL* | F: GCTGGAGGAGTGTTTTTTTGC |
|  | R: AGTTGAACCAAGCAGGTCACA |
| *MGAT1* | F: GCTATTTCCTGGCTTTACATCG |
|  | R: AACCCTTTGCGCTGGCGGAT |
| *DGAT1* | F: TTCCGCCTCTGGGCATT |
|  | R: AGAATCGGCCCACAATCCA |
| *DGAT2* | F: AGTGGCAATGCTATCATCATCGT |
|  | R: TCTTCTGGACCCATCGGCCCCAGGA |
| *CD36* | F: GGGCTGTGATCGGAACT |
|  | R: CTTGGCTAGATAACGAACTCTG |
| *FATP4* | F: GTGATGCCTTTGTGGG |
|  | R: TGTCGTCTGCGGTGAT |
| *β-actin* | F: TGTCCACCTTCCAGCAGATGT |
|  | R: AGCTCAGTAACAGTCCGCCTAGA |
